# Resolution enhancement using plasmonic metamask for wafer-scale photolithography in the far field

**DOI:** 10.1038/srep30476

**Published:** 2016-07-26

**Authors:** Seunghwa Baek, Gumin Kang, Min Kang, Chang-Won Lee, Kyoungsik Kim

**Affiliations:** 1 School of Mechanical Engineering, Yonsei University, 50 Yonsei-ro, Seodaemun-gu, Seoul 03722, Republic of Korea; 2 Display R&D Center, Samsung Display Co. Ltd., Yongin City, Gyeonggi-do 17113, Republic of Korea; 3 Samsung Advanced Institute of Technology, Suwon-si, Gyeonggi-do 16678, Republic of Korea

## Abstract

Resolution enhancement in far-field photolithography is demonstrated using a plasmonic metamask in the proximity regime, in which Fresnel diffraction is dominant. The transverse magnetic component of the diffracted wave from the photomask, which reduces the pattern visibility and lowers the resolution, was successfully controlled by coupling with the anti-symmetric mode of the excited surface plasmon. We obtained a consistently finely-patterned photoresist surface at a distance of up to 15 μm from the mask surface for 3-μm-pitch slits because of conserved field visibility when propagating from the near-field to the proximity regime. We confirmed that sharp edge patterning is indeed possible when using a wafer-scale photomask in the proximity photolithography regime. Our plasmonic metamask method produces cost savings for ultra-large-scale high-density display fabrication by maintaining longer photomask lifetimes and by allowing sufficient tolerance for the distance between the photomask and the photoresist.

Numerous efforts have been made to provide resolution enhancement for photolithography to overcome the diffraction-limited nature of typical diffractive optical systems^1^. The resolution limit in the imaging and projection optics of these systems has been overcome using techniques such as phase shift masks^2^,^3^, optical proximity correction^4^, and off-axis-illumination^5^,^6^. In addition, immersion lithography, in which increased numerical apertures are provided using immersion fluids^7^,^8^, and extreme ultraviolet light sources^9^ are currently being actively developed for use in VLSI technologies. However, these techniques have not yet been fully implemented, especially in large-area applications such as television displays, because the photomasks, which are in contact with the photoresist, suffer from short lifetimes.

The most important optical parameters of projection photolithography in the non-contact regime are the resolution (RES) and the depth of focus (DOF)^10^,^11^. These parameters are defined as 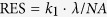 and 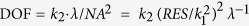, where λ is the irradiation wavelength, *NA* is the numerical aperture of the lens system, and *k*_1_ and *k*_2_ are fabrication factors that depend on the photoresist sensitivity, the photomask complexity, and the lift-off conditions. The fabrication factor *k*_1_ has a higher value under coherent light source irradiation than under incoherent irradiation. While optical systems with higher *NA*s provide better resolution, they also reduce the DOF, and thus restrict the gap separation tolerance between the photomask and the photoresist. Therefore, it is unrealistic to attempt to achieve reduced resolution with enhanced DOF arbitrarily at the same time.

Pendry *et al*. showed that it is possible to focus radiation beyond the diffraction limit using a flat slab made from a double negative refractive index material^12^. The most important concept is projection of the high spatial frequency components in the evanescent wave to the propagating wave using the flat slab made from the negative refractive metamaterial. High spatial frequency components that occur in a normal material and are larger than 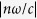, where *n*, *ω*, and *c* are the refractive index, the angular frequency, and the speed of light, respectively, are strongly confined near the material’s boundaries and subsequently decay. One material that can sustain a strong evanescent mode is metal. Because of their negative electric permittivities, metals can sustain high spatial frequency electromagnetic modes in the form of surface plasmons near resonance to beat the diffraction limit^13^,^14^,^15^. The shorter spatial wavelength in the near field allows focusing of the electromagnetic energy and increased field visibility for subwavelength-scale lithographic patterning^16^,^17^,^18^,^19^,^20^,^21^. If the higher spatial frequency components can be controlled or modulated to maintain the spatial field visibility, it would then be possible to achieve ultrahigh-resolution photolithography in the far field.

Unfortunately, surface plasmons do not propagate into the far field, which makes it difficult to apply them to industrial lithography. Because of the rapid damage caused to the photomasks, contact lithography in the near field suffers from a serious bottleneck in terms of the high process costs. In industrial manufacturing systems for large-scale displays, such as those used for televisions and mobile devices, microscale resolution proximity and projection lithography techniques are commonly used. We therefore need to enhance the resolution of far-field lithography processes by manipulating the high spatial frequency components.

In this work, we use a surface plasmon(SP)-assisted photomask with a dielectric spacer to demonstrate far-field photolithography up to 15 μm away from the mask surface with a 1.5-μm-wide slit structure under partially coherent i-line exposure. While the excited SPs are not perfectly coherent, the modulated transverse magnetic (TM) mode of the incident light that is produced via SP excitation provides spectrally-resonant and intensity-controlled directivity for the transmitted light, which then enables high electromagnetic field visibility for formation of fine photoresist structures. We found that the spatial resolution is conserved at any gap distance between the SP-assisted photomask and the photoresist in the range from 3 μm to 50 μm, where Fresnel diffraction is dominant. We also successfully demonstrate that the propagating field visibility that occurs in the proximity regime under focused lamp illumination can also be maintained in the far-field regime.

## Results and Discussion

### Theory of resolution enhancement with SP-assisted mask

**Figure 1:**
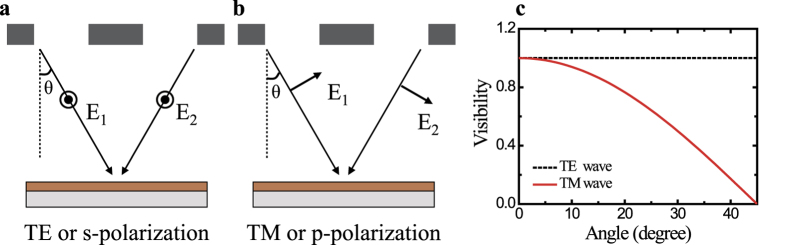
Mirror-symmetric diffracted rays with two polarization states transmitted through a photomask: (**a**) transverse electric (TE) mode and (**b**) transverse magnetic (TM) mode. Here, the angle 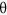 is defined as the angle between the incident wavevector and the diffracted light. This angle is the same as the angle of incidence for a flat photoresist. (**c**) Calculated visibility curve showing the angular dependence of each of the polarization states. The TE wave always has a visibility of 1. However, the TM wave visibility decreases as the angle between the plane waves increases.

In photolithography, the diffracted light that passes through a photomask arrives at a photoresist layer and delivers electromagnetic energy, resulting in formation of the desired interference patterns. The diffracted unpolarized light can, in general, be decomposed into transverse electric (TE, where the electric field is pointing out of the plane of the drawing) and TM (where the electric field is pointing in the plane of the drawing) polarizations, as shown in Fig. 1a,b. Suppose that the two rays have an angle of incidence *θ* with respect to the normal direction. For the TE polarization, the electric fields of the two rays overlap perfectly at all incident angles, and thus the total intensity becomes 
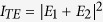
. In contrast, for the TM polarization, the overlap between the two rays decreases as *θ* increases, thus leading to a total intensity of 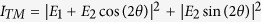
22,23. When *θ* = 0°, *I*_*TM*_ becomes the same as *I*_*TE*_, which signifies the coherent sum of the two fields showing the general property of the lowest diffraction order. When *θ* = 45°, the two electric fields are perpendicular to each other and *I*_*TM*_ becomes a perfectly incoherent sum as 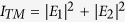. In the general case of two incident plane waves at an angle *θ*,


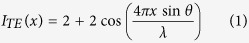



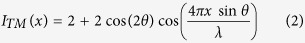


The high diffraction order components have high spatial frequencies, i.e., the larger traveling angle *θ* shown in Fig. 1b.

We consider the visibility, which is defined as 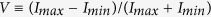, and use it to evaluate the resolution of the photolithography process as a figure of merit. For the TE wave, the visibility is always exactly 1. For the TM wave, however, the visibility depends on the angle of incidence in proportion to cos(2*θ*). As the angle between the incident TM waves increases, the visibility of the resulting image decreases, as shown in Fig. 1c. Therefore, we may consider the TM wave as having a ‘bad’ polarization that reduces the image quality. Note that any structure located parallel to the TE polarization has good lithographic resolution and a good DOF. Therefore, without loss of generality, we aim to increase the visibility of the TM wave.

High spatial frequency components in the TM polarization state under perfectly coherent irradiation can provide super-resolution at specific locations; this is known as the Talbot effect^24^,^25^. However, Talbot lithography requires fine control of the distance between the photomask and the photoresist to ensure good resolution and a good DOF. Additionally, provision of perfectly coherent irradiation using lasers increases both fabrication time and costs for larger area lithography when compared with incoherent lamp irradiation. Our strategy is to suppress the higher diffraction order components of the TM modes using the SP-assisted photomask, which leads to high resolution even in the far-field regime under either partially coherent or incoherent irradiation. By using an appropriate metal, it is possible to resonantly couple the incident light with TM polarization to one of the SP modes. This mode would effectively suppress the TM wave, thus allowing fairly good resolution and a good DOF even when the photoresist is located comparatively far from the photomask. In addition, the tolerance errors of the distance between the photoresist and the photomask can be significant. It is important to maintain the irradiation energy at a level close to that of the SP resonance energy for optimal resolution enhancement.

**Figure 2:**
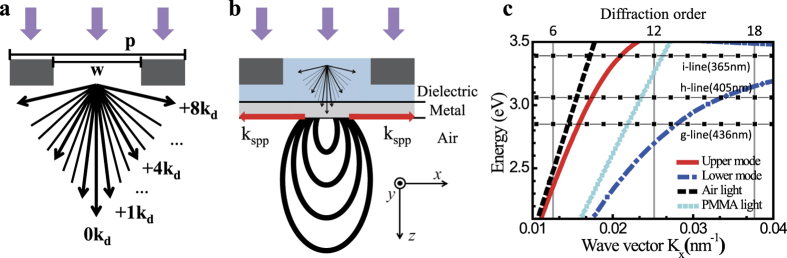
Schematic figures of propagating light transmission through periodic slit apertures with width *w* in the cases of (**a**) the binary photomask and (**b**) the SP-assisted photomask. (**c**) Dispersion relations of (1) Fresnel diffraction modes (black rectangular dots) for three representative irradiation energies, i.e., i-line at 365 nm, h-line at 405 nm, and g-line at 436 nm, (2) SP modes, and (3) propagating modes in the air (black line) and in poly (methyl methacrylate) (PMMA, cyan line). Here, the slit width and the periodicity are 1.5 μm and 3 μm, respectively. In the asymmetric multilayer structure, the anti-symmetric lower SP mode (blue curve) coincides with the higher Fresnel diffraction order modes, whereas the upper SP mode (red curve) coincides with the lower Fresnel diffraction modes. Therefore, appropriate SP excitation allows the control or modulation of the higher diffraction order of the transmitted irradiation after it passes through the photomask.

Consider a slit with width *w* (where pitch = 2*w*), as shown in Fig. 2a. We use the period as a measure of the diffraction order for irradiation at a wavelength of 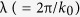. The corresponding TM electromagnetic field can be decomposed into its spatial harmonics of 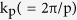, e.g., 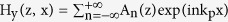, where the diffraction order *n* is a integer that satisfies 
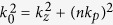
 26. The super-resolving fields at the higher spatial frequencies, where 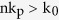, decay exponentially to become evanescent waves. The lower spatial frequency fields, where 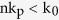, simply propagate. When the 365 nm light is directed through the 1.5-μm-slit-width photomask, the light is transmitted by diffraction order *n*. The arrowed lines in Fig. 2a show the components of the Fresnel diffraction order formed by the propagating far-field light. The high diffraction order components have high spatial frequencies, i.e., a larger traveling angle θ. In the far-field photolithography regime, the modulation of the higher orders of the Fresnel diffraction components strongly affects the photoresist visibility. The red lines in Fig. 2b show the SP waves that are generated by the metal-dielectric layers in the SP-assisted photomask. The higher Fresnel diffraction modes can then be transformed into SP modes that are confined at the metal-dielectric interface. Therefore, the field lobe beyond the SP-assisted photomask becomes increasingly elliptical, as shown by the black curve in Fig. 2b.

Rather than use a projection lens system, which may require more than ten ordinary lenses, we consider the use of a simple additional structure that is composed of a dielectric spacer and a thin metal layer, as shown in Fig. 2b. By coupling the diffracted TM polarized light with the SP that is excited at the metal/air interface^27^, it becomes possible to achieve enhanced persistent resolution in the proximity regime. The red lines at the metal/air interface show the excited SPs and their propagation directions. To provide greater detail, we calculated the SP dispersion curves for a 1.5-μm-wide microscale slit, as shown in Fig. 2c. For an asymmetric poly(methyl methacrylate) (PMMA)/Ag (35 nm)/air structure, two modes emerge: the upper mode (red line, symmetric) and the lower mode (blue line, antisymmetric) [see the Supplementary Information, section 1]. We also draw three horizontal lines that correspond to the energies of the i- (365 nm), h- (405 nm), and g-lines (436 nm) that are commonly used in photolithographic mask aligners. When the energy and the momentum of the SP mode are matched with the corresponding properties of some of the diffracted modes, the incident light is converted into the SP. The higher spatial frequency modes for Fresnel diffraction above this crossing point are all transformed into SP modes, because no propagating mode exists in the air. As a result, the overall field lobe that passes through the SP-assisted photomask becomes increasingly elliptical, as shown by the black curve in Fig. 2b. We find that the SP spatial frequencies for the i-, g-, and h-lines are matched to the diffraction orders of the 10^th^, 8^th^, and 8^th^ modes, respectively, in the case of a 1.5 μm slit width.

### Simulated results of photolithography using SP-assisted metamask

**Figure 3:**
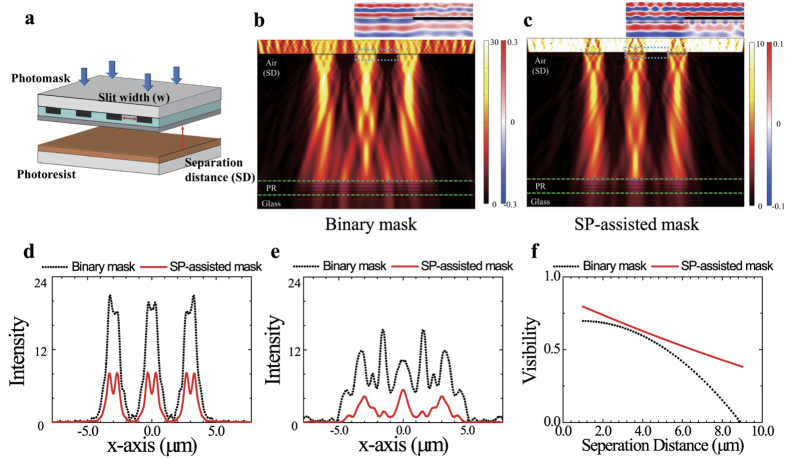
(**a**) Schematic of photolithographic process with slit width *w*, where the separation distance (SD) between the photomask and the photoresist is indicated. Simulated 2D field intensity profiles are shown for (**b**) the binary mask and (**c**) the SP-assisted mask in the case where *w* = 1.5 μm and SD = 10 μm. The field intensity profiles are calculated for the sum of all the irradiation lines for the i-line, h-line and g-line with TM polarization. The top figures shown in (**b**,**c**) are the EM field profiles at the nearby photomasks. The intensity profiles in the photoresist are shown for (**d**) SD = 1 μm and (**e**) SD = 10 μm. When SD = 1 μm, both the binary photomask and the SP-assisted photomask show good intensity visibilities along the *x*-axis. The intensity visibilities from the binary photomask become poor when SD = 10 μm because of interference effects, particularly at *x* = 1.5 μm, while the SP-assisted photomask allows relatively good field intensity visibilities. (**f**) Visibility as a function of SD. The binary mask shows a more abrupt reduction in visibility when compared with that of the SP-assisted photomask. The latter mask also provides better resolution in the proximity regime than the binary photomask.

Figure 3a shows a schematic of photolithographic processing with slit width *w*, where the separation distance (SD) between the photomask and the photoresist is indicated. We simulated the field intensity profiles of the light when transmitted through a binary mask (Fig. 3b) and the SP-assisted photomask (Fig. 3c). We used the COMSOL Multi-physics package with Rakic’s optical property^28^ and a photomask with three open slits with a 3 μm pitch, 1.5 μm slit widths, and a 10 μm SD. Here, the 2D intensity field profiles are shown for the sum of all the irradiation lines for the i-line, h-line and g-line wavelengths with TM polarizations. We note that the Talbot-like effect in this simulation occurs because of the coherence of the light source. In the experiments, we were unable to detect any Talbot effect because we used an incoherent mercury lamp as the irradiation source. The top figures in Fig. 3b,c show the summed electromagnetic fields near the photomask. We observe from these intensity profiles that the SP-assisted mask produces fewer interference fringes at the photoresist plane, thus implying good lithographic quality. In addition, the electromagnetic wave along the surface of the plasmonic photomask indicates that the SP wave is indeed generated at the interface between the silver layer and the PMMA layer.

Figure 3d,e show the cross-sectional intensity profiles for SD values of 1 μm and 10 μm, respectively. When SD = 1 μm, the two masks show similarly good visibilities, as expected from the contact regime. However, when SD = 10 μm, the SP mask shows much better visibility than the binary mask. In terms of their intensity profiles, the SP mask intensity for the diffracted light is also reduced almost by half. It is inevitable that reduced transmission will be obtained because our plasmonic mask suffers from ohmic losses due to the finite conductivity of the metal and radiation losses due to its imperfect surface structure. However, the image quality is dependent on the contrast (visibility) rather than the absolute value of the intensity on the photoresist. We therefore plotted graphs of simulated visibility versus SD for the binary and SP masks, as shown in Fig. 3f. It is conventionally accepted that the patterns are distinguishable if the visibility is higher than 0.4. The binary mask has visibility of >0.4 up to SD = 5 μm, and thus does not work well out of its normal range. The SP mask shows visibility of >0.4 up to SD = 10 μm, and thus we can use this mask for reliable proximity lithography.

**Figure 4:**
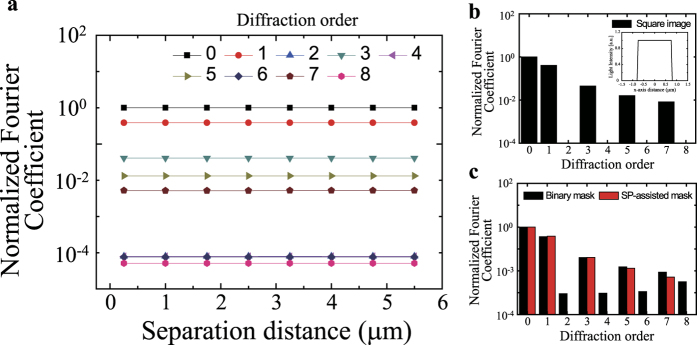
(**a**) Calculated normalized Fourier coefficients for each order of the diffraction components obtained from the intensity profile of the SP-assisted photomask as a function of SD. (**b**) Normalized Fourier coefficients of an ideal square field profile (shown in the inset) calculated for image quality comparison. (**c**) Normalized Fourier coefficients as a function of diffraction order for the binary mask (black) and the SP-assisted mask (red).

From the field profiles, we extracted each of the diffraction order propagation component coefficients at the SP-assisted mask as a function of the SD (Fig. 4a). For diffraction orders of less than 8, the light propagates, and thus the corresponding coefficients are constant over the SD. For diffraction orders of >8, the light decays exponentially. Therefore, propagating light with diffraction orders of <8 is highly effective for image formation in the photoresist in the far-field regime [see Supplementary Information, section 2].

For quantitative estimation of the lithography quality, we calculated the normalized Fourier coefficient of an ideal field shape, i.e., a square electromagnetic field profile (as shown in Fig. 4b). The coefficient only has a significant value for odd numbers. For the SP-assisted mask, the coefficients of the propagating light components with n = 2, 4, 6, 7 and 8 are much smaller than the corresponding values for the binary mask (Fig. 4c). The components of the SP mask’s coefficient are very similar to those of the ideal square field profile.

### Experimental results for SP-assisted photolithography

**Figure 5:**
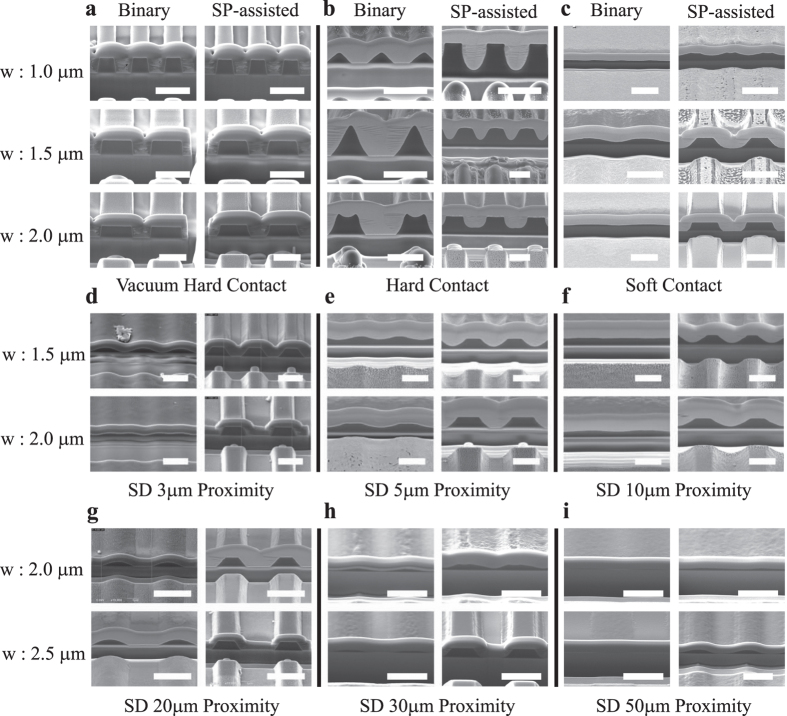
Scanning electron microscopy (SEM) images of patterned photoresists on 6-inch substrates obtained using various binary masks and SP-assisted masks. (**a–c**) Patterned photoresists in various contact regimes. The distance between the photoresist and the photomask can be controlled using the pressure applied to the photomask. Vacuum hard contact, hard contact, and soft contact correspond to contact pressures of 3 N/cm^2^, 0.22 N/cm^2^, and 0.05 N/cm^2^, respectively. In the vacuum hard contact regime, both the binary mask and the SP-assisted mask produce high photoresist visibilities for slit widths of 1 μm, 1.5 μm, and 2 μm. However, in the hard contact and soft contact regimes, the SP-assisted photomask produces better photoresist patterns for any slit width (the scale bar represents 2 μm). (**d–i**) Patterned photoresists in proximity regimes for SD = 3 μm, 5 μm, 10 μm, 20 μm, 30 μm, and 50 μm, respectively. Regardless of the slit width values from 1.5–2.5 μm, the SP-assisted masks produce better photoresist patterns (the scale bar represents 3 μm).

To characterize the far-field photolithography process when using the SP-assisted mask, we experimentally compared photolithographic patterning results with and without the plasmonic structure in the photomask in both contact and proximity modes. The SD between the photomask and the photoresist (PR) wafer was controlled such that it varied from contact modes (vacuum-hard, hard, and soft contacts) to proximity modes with SD = 3 μm, 5 μm, 10 μm, 20 μm, 25 μm, and 50 μm, as shown in Fig. 5. The contact pressures were 3 N/cm^2^, 0.22 N/cm^2^, and 0.05 N/cm^2^ for the vacuum-hard, hard, and soft contact modes, respectively. Under the illumination of the mercury lamp (350–450 nm) of the EV620 photo aligner, we determined optimum exposure doses of 90 J/cm^2^ and 300 J/cm^2^ and obtained the required photolithographic patterns for the binary mask and the SP-assisted mask, respectively. Because we use positive PRs, the light-exposed area is developed in each case.

Figure 5a–c show the experimental photolithographic patterning results for the vacuum-hard, hard, and soft contact modes, respectively, when using the 1 μm, 1.5 μm, and 2 μm slit widths and for both the binary and SP-assisted masks in each case. In the vacuum-hard contact mode with 3 N/cm^2^ contact pressure, the SD is intended to have the smallest value and be within the near field range, and thus we obtained high photoresist visibilities from both masks (see Fig. 5a). In the hard mode with 0.22 N/cm^2^ contact pressure, the sharp edges appeared to have collapsed when the slit width of the binary mask was 1 μm or 1.5 μm. In the 2 μm slit width case, the pattern was formed using the upper interference pattern in the upper region. In contrast, patterning using the SP-assisted photomask produces high visibility with sharp edges across all slit width values. In the soft contact mode with contact pressure of 0.05 N/cm^2^, the visibilities are close to 0 for every slit width value. In contrast, the SP-assisted photomask generates edged square shapes for the gratings at all slit width values other than 1 μm.

In the contact mode, we find that there are limits to the possible slit width values that are dependent on the contact pressure and the SD value. The proximity mode, as an alternative, allows us to test the patterning limits for a variety of SD values. Figure 5d–i show the experimental photolithographic patterns at various controlled SDs in the far field for the cases of irradiation of 1.5 μm, 2 μm, and 2.5 μm slit width photomasks in the proximity mode. Figure 5d–f show the 1.5 μm and 2 μm slit width patterns for SDs of 3 μm, 5 μm, and 10 μm, respectively. These patterns are similar to the results that were obtained in the contact mode. As shown in the figures, the binary mask fails to produce either good visibility or sharp edges as the SD value increases. The SP-assisted mask, however, maintains good visibility even with the increased SD values. We also observed smoother edges with the increased SD values. In the case of the 1.5 μm slit width and the 10 μm SD value, the experimental result obtained using the SP-assisted mask almost perfectly reproduces the simulated result. Patterns are not produced for SDs of more than 20 μm for the 1.5 μm slit width. The maximum SD value for the 1.5 μm slit width case is therefore estimated to be 10–15 μm.

Similarly, we performed patterning for the 2 μm and 2.5 μm slit width cases with larger SD values of 20 μm, 30 μm, and 50 μm. The scanning electron microscopy (SEM) images shown in Fig. 5g–i show the patterns for the slit widths of 2 μm and 2.5 μm. The binary mask could not produce nonzero visibility or sharp edges in any of the cases. In contrast, the SP-assisted photomask allows sharp edge patterning up to the SD value of 20 μm in proximity mode for the 2 μm slit width case. For the 2.5 μm slit width, suitable patterning up to the SD value of 30 μm could be obtained when using the SP-assisted photomask.

In summary, we have demonstrated resolution enhancement photolithography in the proximity regime for SD values of up to 15 μm when using the SP-assisted photomask with a 1.5 μm slit width. We calculated the plasmon dispersion relation to determine the closest match to i-line conditions by selecting a 35-nm-thick Ag layer to suppress the higher order TM diffraction modes. As a result, the transmitted wave that passes through the SP-assisted photomask shows high visibility, superb resolution, and sharp edges in the carved photoresist that exceed the visibility and the resolution that were acquired when using the contact mask without SP assistance. We fabricated several lithographic patterns to check the distance-dependent transcription of the field to the photoresist and confirmed that the SP-assisted photomask indeed allows much finer patterning than the binary mask at any SD. We expect that this technology will be readily applicable to photolithography on a much larger scale for large screen displays, with an extremely extended photomask lifetime leading to fabrication cost reduction.

## Methods

### Fabrication of SP-assisted metamask and photolithography method

We fabricated two sets of 6-inch-diameter binary masks consisting of Cr line-space gratings with slit width (*w*) values of 1 μm, 1.5 μm, 2 μm, and 2.5 μm [see Supplementary Information, section 3]. While Cr can support SPs, the corresponding resonance energy lies in the near infrared region and can be neglected in the ultraviolet spectrum. Using one of these masks, we fabricated SP-assisted photomasks by depositing a 40-nm-thick PMMA layer and a 35-nm-thick Ag layer on the Cr gratings of the binary mask. The PMMA layer thickness was controlled by inductively-coupled plasma (ICP) ashing (ALA-0601E, AMS) after spin coating. The Cr and Ag layers were deposited using the Evaporator UEE (Ultech). We then spin-coated the PR (positive PR, DPR-i7000) on a quartz substrate. Using the EV620 photomask aligner (EVG), we varied the SD between the photomasks and the PR wafer from a hard contact mode to a proximity mode in the far-field regime. The irradiation source was a mercury lamp (operating at 350–450 nm), with the light being passed through a circular hole to maintain partial coherence. We controlled the exposure dose and obtained photolithographic patterns for both the binary mask and the SP-assisted mask.

### Characterization of photolithographic profiles

The cross-sectional images of the PR patterns were inspected using a dual electron beam system, which provides both a focused ion beam (FIB) column and a SEM column (FEI Nova 200 Nanolab). The FIB was used to mill the patterns using gallium ions (Ga+) to reveal the cross-sections of the samples. The SEM, with a tilted angle of 52° with respect to the FIB direction, then captured the profiles of the PR patterns.

## Additional Information

**How to cite this article**: Baek, S. *et al*. Resolution enhancement using plasmonic metamask for wafer-scale photolithography in the far field. *Sci. Rep*. **6**, 30476; doi: 10.1038/srep30476 (2016).

## Supplementary Material

Supplementary Information
