# Supplementary material for: Resolution enhancement using plasmonic metamask for wafer-scale photolithography in the far field
Source: Sci Rep. 2016 Jul 26;6:30476. doi: 10.1038/srep30476 (PMC4960539; doi:10.1038/srep30476)
Supplement: Supplementary Information [file srep30476-s1.pdf]

Supplementary Information

Resolution enhancement using plasmonic metamask for wafer-scale photolithography in the far field

Seunghwa Baek[1], Gumin Kang[1], Min Kang[2], Chang-Won Lee[3*], and Kyoungsik Kim[1*]

[1] School of Mechanical Engineering, Yonsei University, 50 Yonsei-ro, Seodaemun-gu, Seoul 03722, Republic of Korea.

[2] Display R&D Center, Samsung Display Co. Ltd., Yongin-City, Gyeonggi-Do, 17113, Republic of Korea.

[3] Samsung Advanced Institute of Technology, Suwon-si, Gyeonggi-do 16678, Republic of Korea.

[*]Correspondence and requests for materials should be addressed to K.K. (e-mail: kks@yonsei.ac.kr) or C.-W.L. (email: cwlee42@gmail.com).

S1. Spatial Fourier expansion of incident irradiation and the dispersion relation of the SP formed in the metamask

Consider a binary mask with a period of d. When the incident illuminated light is normal to the grating, the electromagnetic (EM) field can also be decomposed into its spatial harmonics of mk_d (m is integer). The transmitted TE polarized EM fields in the free space are,

$$E_{TE}(x,y,z) = \hat{y}\sum_{m=-\infty}^{\infty} T_m \exp[i(mk_d x + k_z z)] \quad (1)$$

$$H_{TE}(x,y,z) = k^{-1}\sum_{m=-\infty}^{\infty} T_m(-k_z\hat{x} + m\,k_d\hat{z}) \times \exp[i(mk_d x + k_z z)] \quad (2)$$

where the incident field is given by $E(x,y,z) = \hat{y}E_0(ik_0 z)$. The TM polarize light, incident field can be written H(x,y,z) = $\hat{y}H_0(ik_0 z)$, where

$$E_{TM}(x,y,z) = k^{-1}\sum_{m=-\infty}^{\infty} L_m(k_z\hat{x} - m\,k_d\hat{z}) \times \exp[i(mk_d x + k_z z)] \quad (3)$$

$$H_{TM}(x,y,z) = \hat{y}\sum_{m=-\infty}^{\infty} L_m \exp[i(mk_d x + k_z z)] \quad (4)$$

In eqs. (1) - (4), T_m, L_m are coefficients of representing the complex amplitudes of the diffraction orders generated by the slit. When $mk_d = 2\pi m/d$,

$$k_z = k_0 - mk_d \quad (5)$$

is the vector important for constructing imaging. As shown in Figure2a in the maintext, propagation wave vector (k_z) has a different angle to the free space representing propagation spatial frequency fields. Here we assume d is 3 μm. The Fourier coefficient, L_m, can be calculated by following integral,

$$L_m = d^{-1}\int_0^d H_{TM}(x,0,z)\exp(imk_d x)\,dx \quad (6)$$

For SP-assisted photomask structure, we consider the waves guided by asymmetric structures that a silver slab of thickness h (medium $2\,(m)$: $\varepsilon_m = \varepsilon_{m,R} + i\varepsilon_{m,I}$) is sandwiched by PMMA (medium 1 : ε_1) and air (medium 3 : ε_3), as given in Figure 2b. The surface plasmon (SP) waves propagating in the i-th medium $(i = 1, m, 3)$ are TM waves and can be described by the magnetic field propagating along the surface of x-axis, $\vec{H}^{(i)} = \hat{j}H_y^{(i)} = \hat{j}H_0 f^{(i)}(z)\exp[i(\omega t - \beta x)]$, where β is the complex propagation constant along the

surface and $f^{(i)}(z)$ represents the depth dependence if H_0 is a normalization constant. The electric field components are derived as $E_x^{(i)} = \frac{i}{\omega\varepsilon_0\varepsilon}\frac{\partial H_y^{(i)}}{\partial z}$, $E_z^{(i)} = -\frac{\beta}{\omega\varepsilon_0\varepsilon} H_y^{(i)}$ and the depth distributions are calculated as $f^{(1)}(z) = e^{S_1 z}$ $(z < 0)$, $f^{(m)}(z) = \cosh(S_2 z) + \frac{S_1\varepsilon_m}{S_2\varepsilon_1}\sinh(S_2 z)$ $(0 < z < h)$, and $f^{(3)}(z) = \left[\cosh(S_2 h) + \frac{S_1\varepsilon_m}{S_2\varepsilon_1}\sinh(S_2 h)\right] e^{-S_3(z-h)}$ $(z > h)$, where S_1, S_2, and S_3 are complex longitudinal wave numbers as $S_1^2 = \beta^2 - \varepsilon_1 k_0^2$, $S_2^2 = \beta^2 - \varepsilon_m k_0^2$, and $S_3^2 = \beta^2 - \varepsilon_3 k_0^2$, for a given $k_0 = 2\pi/\lambda$.[1]

From the continuity condition of the tangential electric field at the interfaces, we obtain the dispersion relation as following,

$$\tanh(S_2 h)\,(\varepsilon_1\varepsilon_3 S_2^2 + \varepsilon_m^2 S_1 S_3) + [S_2(\varepsilon_1 S_3 + \varepsilon_3 S_1)\varepsilon_m] = 0 \tag{7}$$

Eq. (7) were analyzed using the Ag optical constants of Rakic.[2] The solutions can be categorized depending on the signs of S_1 and S_3. When both signs of S_1 and S_3 are negative, no solutions exist. If the signs of S_1 and S_3 are different leaky modes are formed. For $S_1 > 0, S_3 < 0$, the solution of Eq. (7) becomes symmetric leaky mode transmitting the mask. For $S_1 > 0$ and $S_3 > 0$, there are dispersion curves for two bound modes. These modes show symmetric or anti-symmetric field distributions with respect to the sign changes of the H-field across the Ag layer. The anti-symmetric mode has lower energy than that of the symmetric mode. The layer structure of PMMA/Ag/Air as well as the lossy nature of a Ag film allow strong coupling between the transmitted wave and these surface plasmon bound mode when energy and momentum match with each other. Figure 2c in the maintext shows the dispersion curves of the two bounded modes as well as Fresnel diffraction modes (black rectangular dots) for three representative irradiation energies. (i-line at 365 nm, h-line at 405 nm, and g-line at 436 nm)

S2. Normalized Fourier coefficients as a function of SD

Fourier coefficients of each order of the diffraction components are normalized with respect to zeroth order coefficients. We plot them for the binary mask (a) and for the SP-assisted photomask (b) as a function of the SD. The Fourier coefficients of the propagated light reaching the plane of photoresist are exponentially decayed for more than 9th orders.

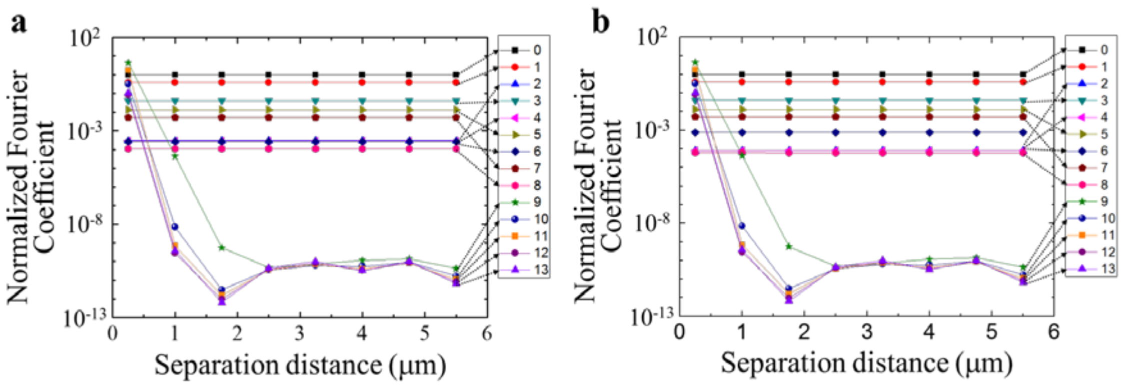

S3. Material property for electromagnetic simulations (COMSOL, finite element method)

Materials	Permittivity values used		
	365 nm (i-line)	405 nm (h-line)	436 nm (g-line)
Glass	2.3787	2.3612	2.3508
Chrome	-3.7057+9.9861i	-4.1328+11.750i	-4.4436+13.639i
PMMA	2.332	2.306	2.292
Silver	-2.1322+0.5064i	-3.7962+0.5433i	-5.0667+0.6104i

S4. 6-inch diameter binary mask and SP-assisted mask

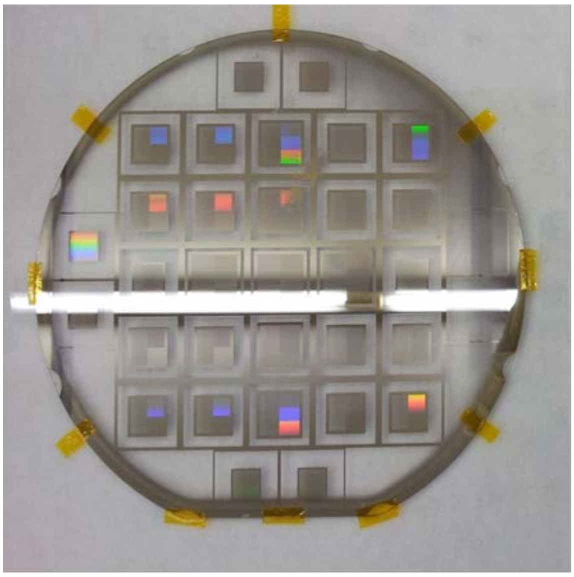

Binary mask

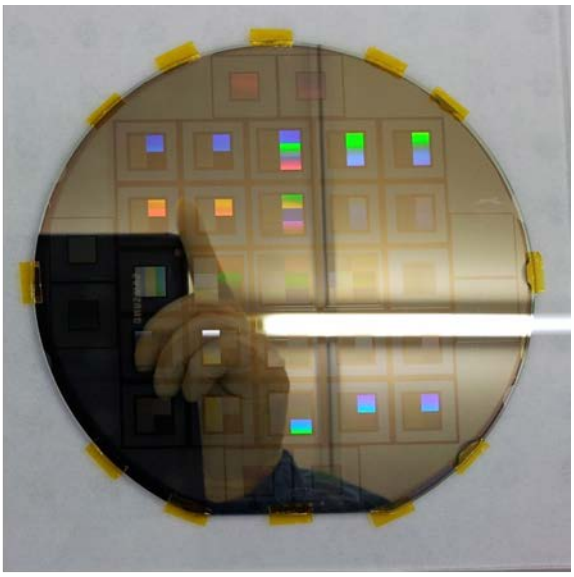

SP-assisted mask

References

(1) Burke J., Stegeman G., Tamir T., Surface-polariton-like waves guided by thin, lossy metal films. Physical Review B 1986; **33**: 5186.

(2) Rakić A.D., Djurišić A.B., Elazar J.M., Majewski M.L., Optical properties of metallic films for vertical-cavity optoelectronic devices. Applied optics 1998; **37**: 5271-5283.
